# The reflex of the past – the role of youth victimisation trauma on the interpersonal reactivity

**DOI:** 10.1080/07853890.2021.1896192

**Published:** 2021-09-28

**Authors:** Ana R. Fonseca, Raquel M. Fernandes, Telma C. Almeida

**Affiliations:** a Instituto Universitário Egas Moniz (IUEM), Egas Moniz Cooperativa de Ensino Superior, Caparica, Portugal; b Laboratório de Psicologia Egas Moniz (LabPSI-EM), Centro de Investigação Interdisciplinar Egas Moniz (CiiEM), Egas Moniz Cooperativa de Ensino Superior, Caparica, Portugal

## Abstract

**Introduction:**

All types of violence perpetrated to a child or adolescent affect their future interpersonal relationships and may also have implications on mental health, academic, and professional performance [1]. About half of the people who have suffered at least one type of victimisation have shown behavioural changes [2]. The main objective of the current study is to analyse the relationship between youth victimisation trauma and the interpersonal reactivity (empathy) in adulthood.

**Materials and methods:**

The sample was composed by 198 Portuguese adults between 18 and 69 years old (*M* = 26.9, *SD* = 11.7), the majority was single (*n* = 168, 84.8*%*), and completed higher education (*n* = 96, 48.5%). Participants answered online to a sociodemographic questionnaire, the Childhood Trauma Questionnaire (CTQ) [3], and the Interpersonal Reactivity Index (IRI) [4]. The study was conducted following the ethical principles and was approved by the university institutional review board and ethics committee.

**Results:**

There was a positive association between the total score of child abuse and the Personal Distress (*r* = 0.223, *p* =.002) and Fantasy (*r* = 0.160, *p* = 025) in adulthood. Personal Distress was positively correlated with Emotional Neglect (*r* = 0.191, *p* = .007), with Physical Abuse (*r =* 0.235, *p* = 0.01), and Physical Neglect (*r=* 0.252*, p <* 0.001). Fantasy was also positively correlated with Emotional Abuse (*r =* 0.182, *p* = .010) and Sexual Abuse (*r =* 0.160, *p* =.024). The total score of the IRI showed a positive correlation with Physical Abuse (*r =* 0.174, *p =* .014) and Physical Neglect (*r =* 0.167, *p* = .019). There was a negative correlation between Empathic Concern and Emotional Neglect (*r* = −0.153, *p* = .032). There was also positive statistically significant correlations between the total score of the CTQ and the occurrence of Emotional Abuse (*r =* 0.843, *p* < 0.001), Emotional Neglect (*r =* 0.793 *p* < 0.001), Physical Abuse (*r =* 0.641, *p* < 0.001) and Physical Neglect (*r* = 0.739, *p* < 0.001).

**Discussion and conclusions:**

This study showed the relationship between youth victimisation trauma and the interpersonal reactivity (empathy), namely, with the increase of personal distress and the ability to place themselves in fictitious situations related to the fantasy in adulthood. Our research also provides a tendency to fall victim to various types of childhood abuse, such as emotional abuse, emotional neglect, physical abuse, physical neglect, and sexual abuse [5]. According to some studies, when children suffer from neglect, they tend to show future problems on interpersonal relationships, more specifically in the capacity to empathise with others [6], and this study corroborates those outcomes.
